# Clinical Diagnostic Clues in Crohn's Disease: A 41-Year Experience

**DOI:** 10.5402/2012/285475

**Published:** 2012-11-19

**Authors:** C. Quintana, L. Galleguillos, E. Benavides, J. C. Quintana, A. Zúñiga, I. Duarte, J. Klaassen, M. Kolbach, R. M. Soto, S. Iacobelli, M. Álvarez, A. O'Brien

**Affiliations:** ^1^Facultad de Medicina, Pontificia Universidad Catolica de Chile, 8330024 Santiago, Chile; ^2^Facultad de Medicina, Universidad de los Andes, 7620001 Santiago, Chile; ^3^Facultad de Medicina, Universidad de Chile, 8380453 Santiago, Chile

## Abstract

Determining the diagnosis of Crohn's disease has been highly difficult mainly during the first years of this study carried out at the Pontificia Universidad Catolica (PUC) Clinical Hospital. For instance, it has been frequently confused with Irritable bowel syndrome and sometimes misdiagnosed as ulcerative colitis, infectious colitis or enterocolitis, intestinal lymphoma, or coeliac disease. Consequently, it seems advisable to characterize what the most relevant clinical features are, in order to establish a clear concept of Crohn's disease. This difficulty may still be a problem at other medical centers in developing countries. Thus, sharing this information may contribute to a better understanding of this disease. Based on the clinical experience gained between 1963 and 2004 and reported herein, the main clinical characteristics of the disease are long-lasting day and night abdominal pain, which becomes more intense after eating and diarrhoea, sometimes associated to a mass in the abdomen, anal lesions, and other additional digestive and nondigestive clinical features. Nevertheless, the main aim of this work has been the following: is it possible to make, in an early stage, the diagnosis of Crohn's disease with a high degree of certainty exclusively with clinical data?

## 1. Introduction

The term “regional ileitis” was described for the first time by Crohn et al. in 1932 [1, 2] as a new pathologic entity characterized by subacute or chronic necrotizing and cicatrizing inflammation of the terminal portion or portions of the small intestine. Over time, it was clear that the disease was much more complex than that. In fact, in the subsequent years, there were many attempts to characterize this new disease in more descriptive terms like terminal ileitis, regional enteritis, and “ileojejunitis”. In the author's opinion, there is no good current definition of the disease that encompasses all the possible clinical manifestations of this entity. Given the widespread nature of the disease, the symptoms are not limited to the gastrointestinal system, in fact, it may compromise joints, skin, eyes, and other systems. The clinical picture of Crohn's disease may be confused with other ailments, such as ulcerative colitis, infectious colitis or enterocolitis, irritable bowel disease, colon cancer, coeliac disease, intestinal lymphoma, and rheumatoid arthritis.

In 1967, Scadding wrote that, according to general experience, carefully describing the clinical features and pathological data of a disease is fundamental in order to properly identify it and that after a variable observation period, it is possible to formulate the essential facts that characterize it [3]. The aim of this work was precisely to characterize Crohn's disease in our population, primarily on clinical grounds, with a reasonable degree of certainty. Without a doubt, this was the position of this study from the beginning, since Crohn's disease was, by that time, almost unknown in Chile in 1963.

## 2. Patients and Methods

This study contains clinical data collected from patients diagnosed between July 1963 and August 2004 at the PUC Clinical Hospital. In general, the patients had suffered from long-lasting abdominal discomfort for more than three years. Infectious and tumorous pathologies were ruled out for these patients. The diagnosis for the entire sample group was confirmed by endoscopy, histology, and/or imaging studies. Patients whose data could not be obtained were excluded from the study.

Patients' histories were also studied, including emotional events and possible extradigestive symptoms, habits, surgical procedures and family history. After this work-up, laboratory tests and other specific procedures, were performed in order to confirm or discard the presumed diagnosis. Statistical analyses were carried out with the Student's *t*-test and the chi square test.

## 3. Results

We studied 309 patients, 142 males (46%) and 167 females (54%). The ethnic origin of the patients was predominantly Hispanic-Amerindian (see Table 1). According to data of the International Organization for Migrations, Caucasian not Hispanic descendant population in Chile corresponds to about 1.600.000 inhabitants, arabian to 800.000, jew to 75000. The 2002 chilean census gives to Chile a total population of 15.116.435 and a mapuche population of 604.349. 

Mean age at consultation was 47.4 ± 14. Nevertheless, the mean age of symptom onset was 35 ± 12.5. Mean diagnostic delay before 1995 was 5.5 years and 4.5 years between 1996 and 2004. However, this difference was not significant (*P*≻0.05). 

**Figure 1:**
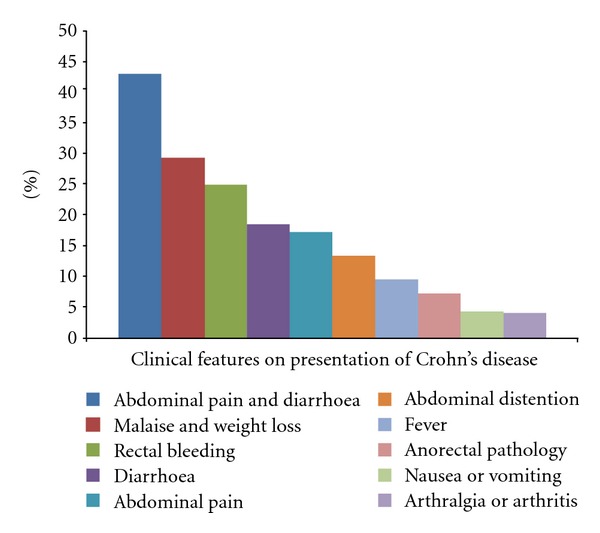


Their symptomatology consisted of abdominal pain and/or diarrhoea (78%), aphthous or ulcerative stomatitis and/or skin involvement (42%). Arthralgia and arthritis (30.7%), weight loss (29%), intestinal bleeding (25%), depression (15%), and anxiety (6.2%) (Figure 1) were also included.

Concerning patient and family history, the following data was recorded: about 85% of the patients have had abdominal surgical procedures (Table 2), 4% had relatives with Crohn's disease, and 8% with Ulcerative Colitis. Interestingly, the occurrence of psoriasis was frequent in the patients' relatives.

Concerning the patients' physical signs, the following data was drawn: 36.2% of the patients had a palpable abdominal mass and 13.9% had anal and perianal lesions (see Table 3); 62% of the patients had a normal body mass index, of which only 16% was below 18.5 kg/m^2^ and, surprisingly, 22% was in the overweight range.

Tables 4, 5, and 6 summarize the rheumatologic, dermatologic, and ocular manifestations of Crohn's disease, which are similar to what has been described elsewhere in the literature [4, 5]. Nevertheless, it is important to emphasize the concomitant suffering of arthralgia or arthritis in 30.7% of the patients, as well as the detection of rosacea and psoriasis in 6.4% of the ample population during the symptom onset stage.

The disease appeared predominantly as ileitis and/or colitis in 84% of the patients (Table 7), and there was a major trend towards the inflammatory mode (in 72% of the patients). The minority had fistulas (15%) or stenosis (13%). During one year of patient follow-up, 24% suffered an exacerbation of disease activity. It might be interesting to note that a statistical correlation between this fact and clubbing was also found (*P*≺0.01).

The main tools that were used to confirm the diagnosis were surgical and endoscopic biopsies in 45% of the patients, endoscopy in 37%, and radiologic studies in 28%. Most patients showed characteristic image findings, and histological samples were compatible with the disease. Regarding laboratory tests, anaemia (defined as haemoglobin ≤ 10 g/dL) was detected in 12.3% of the patients, ESR ≥ 30 mm/hour in 18%, platelets count > 400.000/mm^3^ in 35.9%, and albumin ≤ 3.0 g/dL in 3.2%. In the 61 last patients studied, CRP was measured, and in 48 (78.7%) of them, abnormal high values were detected. 

**Figure 2:**
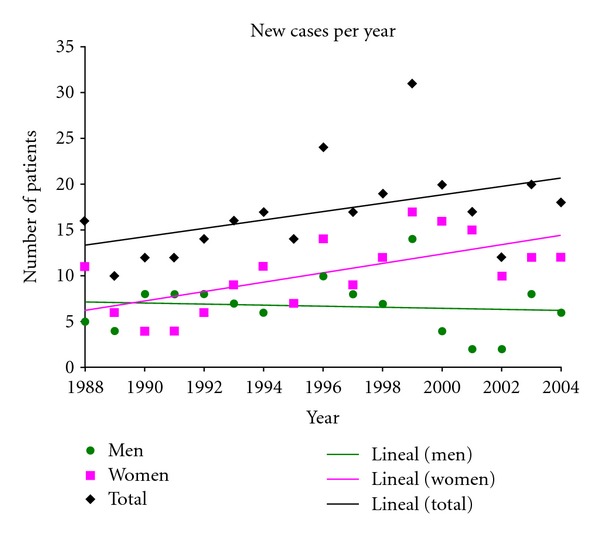


Finally, an increasing trend of new patients suffering from Crohn's disease was observed in the PUC Clinical Hospital (Figure 2), but a significant difference between the periods of 1988–1996 and 1997–2004 (*P*≻0.05) was not found. Nevertheless, there has been an evident increase of new cases after 2004. In fact, in the period 2005–2011, our institution has attended more than 400 new patients, which are not included in the present manuscript because the main author of this work was no longer involved in the clinical study of these patients. 

## 4. Discussion

The fundamental importance of clinical symptoms and physical signs is highlighted in this report, because it was noted at the beginning of this study that earlier diagnosis could be achieved with accurate clinical observation.

There has been a rather slow development of the definition of Crohn's disease since its first pivotal description, almost eighty years ago [1]. After that, it has been necessary to establish a satisfactory empirical clinical definition [6, 7]. Despite the efforts to achieve this goal, there are still other clinical features for which precise criteria are yet to be unveiled. For instance, about 8% of recent patients diagnosed and operated on the PUC Clinical Hospital for Ulcerative Colitis finally ended up being diagnosed for Crohn's disease [8]. 

As far as clinical manifestations of the disease are concerned, it is crucial to stress the importance of the abdominal physical examination, because what is generally denominated as an abdominal mass, either painful or not, may have a variety of tactile representations: hard, soft, well, or poorly defined, and sometimes elongated as a rope or an eel and sometimes subtly detected. In fact, the latter corresponds to an inflamed intestinal segment that in most cases involves the peritoneal and lymphatic adjacent tissues. Although this is not a specific feature of the disease, it may be a very useful clue in the diagnosis, mainly if it is associated with other clinical manifestations of this illness.

Anal and perianal lesions (fissures, fistulas, or abscesses) are also rather frequent in Crohn's disease. According to the records compiled for this study, 14.7% of the patients suffered these complications before or on the date of the diagnosis; unfortunately, sometimes they were interpreted to be “hemorrhoid complications”. In fact, it is interesting to mention that Lockart-Mummery reported that one-fourth of the patients had anal lesions as the first clinical manifestation of Crohn's disease [9].

Arthralgia and arthritis were common findings in the patients of this study, of which 12.3% suffered from peripheral arthritis and 5.8% from sacroiliitis/ankylosing spondylitis, of which, in turn, 2.2% had arthritis before the digestive manifestations of the disease. In comparison, according to Orchard and Jewell in their series of 1409 patients who suffered from inflammatory bowel disease, the prevalence of polyarthritis in 387 patients with Crohn's disease was 12.4% and that of arthralgia was 14.3% [10], while Van Patter et al. reported only a 4.5% prevalence of arthritis [11]^.^ The data exposed herein is, thus, similar to that by Orchard and Jewell. Regarding axial arthritis, the published prevalence appears to be unexpectedly wide, that is, from 1% to 6% [10]. It may also be interesting to mention that pain in the sacroiliac area was a common finding in the patients included in this study, despite the apparent normality seen through imaging results focusing on sacroiliac junctures.

Concerning the dermatologic manifestations of the disease, the most frequent one found in this study was erythema nodosum, which most of the time coincided with a crisis of the disease. However, it sometimes may precede the digestive features of the illness. In fact, one of the patients of this study suffered from erythema nodosum several years before perceiving digestive symptoms. Other features that are likely to be associated with the disease are worthy of being mentioned, including dermatologic abnormalities, such as rosacea that occurred in 3.2% of the patients. Unfortunately, there was no data related to the prevalence of rosacea in this patient population, but data from the UK indicating a 1.8% prevalence should be considered. The frequency of psoriasis among the patients included in this study was 3.2%, which is slightly more than what has been found in the general population elsewhere (2-3%) [12]. It is also less than what has been published regarding Crohn's disease patients in the UK, where 3.97% suffered from psoriasis compared to 2.21% in control subjects [13]. Other publications also demonstrate the association of Crohn's disease and psoriasis [14, 15]. It may be interesting to point out that the frequency of rosacea among the patients observed in this study is the same as that of psoriasis. The increased occurrence of psoriasis has also been observed in relatives of Crohn's disease patients [16], as confirmed herein. Moreover, the same trend has also been observed for rosacea. 

Many ocular complications have been described for Crohn's disease, with an overall rate between 1% and 11% [17, 18], being 10.5% the frequency observed in the patients included in this study. The most common condition was episcleritis, which was observed in 5.8% of the patients. Apart from the latter, the data informed herein are similar to what has been published in the literature [17, 18]. Furthermore, it may be interesting to mention that this patient population included a case with bilateral dacryocystitis, complicated with prominent exophthalmos.

Physical examination may detect clubbing at the early stage of the disease [19, 20]. However, this feature is not mentioned in the medical literature in relation to the severity of the disease. In this study, a positive statistic correlation was found between clubbing and the clinical severity of Crohn's disease, as mentioned above. Therefore, physical manifestations should be considered in order to decide whether a more vigorous therapy approach should be applied in such patients. Moreover, onychomycosis is described as a possible clinical association [21]. 

Appendectomy has been reported to be a risk factor for clinical manifestations of Crohn's disease [22]. There are several hypotheses concerning its mechanism; however, it is still yet to be known [23]. According to these studies, 23% of our patients had their appendix resected. Unfortunately, we do not have precise data of most of the surgical procedures because they were performed elsewhere. Moreover, the majority underwent several other abdominal interventions as well, which could place abdominal surgery in the risk factor category. It is also possible that this rather large number of surgical procedures may be, to a certain extent, the consequence of a previous erroneous diagnosis.

In Chile, the prevalence of tuberculosis (TBC) has steadily decreased during the past 30 years. In 1989, the prevalence of TBC was 52.2/100,000 inhabitants, and in 2005 the prevalence was 18.1/100,000 inhabitants [24]. Regarding intestinal tuberculosis, there is no data available in our country, because its incidence is very low. In fact, in our medical centre, it has been an uncommon manifestation of TBC. Furthermore, the authors have seen no more than 20 patients in almost 50 years.

Notwithstanding the effort placed in studying these patients and the use of new diagnostic tools, the delay in determining the final diagnosis has not essentially changed along these years. According to the results, a significant number of patients were treated for irritable bowel disease for a long period of time before inflammatory bowel disease was suspected in spite of the incorporation of other diagnostic tools, such as CT and MR scanning techniques in 1987 and 1996, respectively, and colonoscopy in 1978. However, the progressive recognizance of the importance of Crohn's disease in our country plus the acquisition of new diagnostic procedures, such as enteroscopy and enteric exploration with capsules, we hope, is going to shorten the delay mentioned above. Moreover, we think that there is a true difficulty in suspecting and demonstrating the diagnosis of Crohn's disease, mainly in its initial stages. 

At the beginning of this work, only sulfasalazine and corticoids were available for the management of patients. In 1990 mesalazine oral in enemas and suppositories could be obtained in our country. In 1992 we started treating patients with immunomodulators. By that time we demonstrated that 6-Mecaptopurine had on 18 patients suffering from Crohn's disease a clear positive therapeutic effect [25]. 10 years after, we began administrating infliximab. One of our publications showed the benefic effect of this drug on 12 patients; in effect, after one week of treatment, the CDAI fell from 357 ± 62  to  138 ± 122  score  points  *P* < 0.005  [26]. The surgical treatment has developed under clear rules since 1978 [27, 28]. In 2008, a publication described the results and follow-up of ileum-caecum resection in Crohn's disease [29]. The main indication for surgery was intestinal obstruction (75%), minor indications were enteric fistulae and lower intestinal bleeding. 14.3% of the patients relapsed (mean time 63 months after the resection above mentioned) and they suffered a second resection of the ileum. 

 This study does not mention other clinical manifestations that are considered to be associated to Crohn's disease, such as psychosocial factors, which have been a focus of attention since the beginning of this study [30, 31]. Indeed, they are very important and constitute a relatively poorly known area [32]. However, the relationship of these factors to the above mentioned information was not included here. Furthermore, pulmonary, vascular, neurological, genitourinary, hepatic and biliary manifestations of this disease appeared in several patients in later stages. 

## 5. Conclusion

The intention of this study was to reach, if possible, a useful clinical concept of Crohn's disease after a careful casuistic analysis. Undoubtedly, this disease has a great number of possible clinical manifestations. Nevertheless, the most frequent and important ones are as follows: day and night abdominal pain, in general refractory to conventional management; diarrhoea, palpable abdominal mass, rheumatologic manifestations, and anal and perianal complications associated to certain extraintestinal clinical signs of the disease.

Secondly, the purpose of exposing this information is that it may be used to suspect the appearance of this disease at an early stage, since initiating its medical management at such phase could yield better results. Although we are not able to assure at the present time and with our data that the clinical data has a high degree of certainty to make the diagnosis, so it is necessary to use technological tools to confirm it. 

Thirdly, this publication could be useful in some medical centers to avoid erratic and useless medical managements, patient anxiety, and unnecessary suffering, as well as possible complications and premature and needless surgery.

## Figures and Tables

**Table 1:** Ethnic origin of the patients.

Ethnic origin	Number of patients	Percentage	% General population
Hispanic-Amerindian	208	67.3	79.6
Caucasian not Hispanic	63	20.4	10.6
Arabian	21	6.8	5.3
Jew	16	5.2	0.5
Mapuche	1	0.3	4.0

**Table 2:** Surgical history.

Surgical history	Number of patients	Percentage
Appendectomy	72	23.3
Bowel resections	70	22.6
Small bowel	23	7.4
Small bowel and colon	26	8.4
Colon	21	6.8
Cholecystectomy	36	11.7
Exploratory laparotomy	36	11.7
Anal fistula or abscess	30	9.7
Anal fissure	8	2.6
Haemorrhoids	9	2.9

**Table 3:** Intestinal physical features in Crohn's disease.

Intestinal physical features	Number of patients	Percentage
Abdominal mass	112	36.2
Fistulae or perianal abscess	46	14.9
Enteric fistulae	40	13
Rectal bleeding	35	11.3
Anal fissure	25	8.1
Haemorrhoids	23	7.4
Anal ulcer	11	3.6
Perirectal abscess	5	1.6
Intra-abdominal abscess	4	1.3
Intestinal obstruction	1	0.3

**Table 4:** Rheumatic features in Crohn's disease.

Rheumatic manifestations	Number of patients	Percentage
Arthralgia	57	18.4
Arthritis	38	12.3
Sacroiliitis/ankylosing spondylitis	18	5.8

**Table 5:** Dermatologic physical features in Crohn's disease.

Dermatologic features	Number of patients	Percentage
Clubbing	27	8.7
Erythema nodosum	26	8.4
Oral ulcers	26	8.4
Aphthous stomatitis	16	5.2
Rosacea	10	3.2
Psoriasis	10	3.2
Pyoderma gangrenosum	6	1.9
Onychomycosis	5	1.6
Seborrhoea	4	1.3

**Table 6:** Ocular manifestations of Crohn's disease.

Ocular manifestations	Number of patients	Percentage
Episcleritis	18	5.8
Uveitis/iritis	6	1.9
Xerophthalmia	3	1
Retinal inflammatory lesions	2	0.6
Keratoconjunctivitis	2	0.6
Dacryocystitis	1	0.3
Ocular pain	1	0.3

**Table 7:** Site of the disease.

Site of the disease	Percentage
Esophagus, stomach, duodenum, and/or jejunum	10.8
Ileitis	30.5
Ileitis and colitis	21.0
Colitis	23.0
Anal-rectal disease	14.7
